# Natural Variation in Resistance to Virus Infection in Dipteran Insects

**DOI:** 10.3390/v10030118

**Published:** 2018-03-09

**Authors:** William H. Palmer, Finny S. Varghese, Ronald P. van Rij

**Affiliations:** 1 Institute of Evolutionary Biology and Centre for Infection, Evolution and Immunity, University of Edinburgh, Edinburgh EH9 3FL UK; W.H.Palmer@sms.ed.ac.uk; 2 Department of Medical Microbiology, Radboud University Medical Center, Radboud Institute for Molecular Life Sciences, P.O. Box 9101, Nijmegen 6500 HB, The Netherlands; Finny.Varghese@radboudumc.nl; 3 Radboud Center for Infectious Diseases, Radboud University Medical Center, Nijmegen 6525 GA, The Netherlands

**Keywords:** *Drosophila* melanogaster, *Aedes aegypti*, vector mosquitoes, RNAi, Toll, IMD, JAK-STAT, microbiota, endogenous viral elements, antiviral defense

## Abstract

The power and ease of *Drosophila* genetics and the medical relevance of mosquito-transmitted viruses have made dipterans important model organisms in antiviral immunology. Studies of virus–host interactions at the molecular and population levels have illuminated determinants of resistance to virus infection. Here, we review the sources and nature of variation in antiviral immunity and virus susceptibility in model dipteran insects, specifically the fruit fly *Drosophila melanogaster* and vector mosquitoes of the genera *Aedes* and *Culex*. We first discuss antiviral immune mechanisms and describe the virus-specificity of these responses. In the following sections, we review genetic and microbiota-dependent variation in antiviral immunity. In the final sections, we explore less well-studied sources of variation, including abiotic factors, sexual dimorphism, infection history, and endogenous viral elements. We borrow from work on other pathogen types and non-dipteran species when it parallels or complements studies in dipterans. Understanding natural variation in virus–host interactions may lead to the identification of novel restriction factors and immune mechanisms and shed light on the molecular determinants of vector competence.

## 1. Introduction

The vast majority of research on invertebrate antiviral immunity has used dipteran systems, including the fruit fly *Drosophila* and the medically important vector mosquitoes of the genera *Culex, Aedes,* and *Anopheles*. These fields have complemented each other, with interest in mosquito-vectored viruses guiding studies in *Drosophila*, and the well-annotated genome and genetic tools of *Drosophila* providing a model to guide laboratory experiments in the less tractable mosquito. The comparison of antiviral processes in these dipterans highlights a conserved yet dynamic immune system, with some antiviral mechanisms conserved from flies to mammals, whereas others have evolved since the expansion of dipterans.

Studies from both fruit flies and mosquitoes have uncovered variation in host–virus interactions. These studies have focused primarily on differences in immune mechanisms between virus species within a host species, or on variation in resistance to a particular virus within or between populations of host species. Insight into virus-specific mechanisms has been gleaned through the use of a diverse set of natural and non-natural viruses in *Drosophila*, and through comparisons of vectored flaviviruses, rhabdoviruses, alphaviruses, and bunyaviruses—part of a larger group referred to as arthropod-borne viruses (arboviruses). These studies reveal a general requirement for antiviral RNAi in dipterans, virus-specific contributions of canonical antimicrobial pathways (such as Toll, Imd, or JAK-STAT), a diverse transcriptional response, and emerging antiviral processes that have yet to be studied in a comparative context (reviewed in [1,2,3,4,5,6,7]). In addition, there is tremendous variation in virus susceptibility between hosts, where the resistance of an individual host is dependent on factors such as genotype, microbiota, infection history, mating status, and the environment. Variation in resistance is further complicated in mosquitoes, where the phenotype of interest is vector competence, the ability of a mosquito to transmit an arbovirus after it had crossed multiple infection barriers (midgut infection barrier (MIB), midgut escape barrier (MEB), salivary gland infection barrier, and salivary gland escape barrier) (reviewed in [8]).

Viral genetic variation, both within and between hosts, is likely to contribute to infection outcomes [9,10]. Arbovirus genetic variation is dynamic within a single host, and is shaped by natural selection and population bottlenecks and expansions at each infection barrier [11,12,13,14]. In some cases, viral variants affecting vector competence have spread through a population, leading to outbreaks of arboviral disease in these regions. For example, single amino acid substitutions in Venezuelan equine encephalitis virus (VEEV) and Chikungunya virus (CHIKV) envelope proteins are associated with increased infectivity in *Ochlerotatus taeniorhynchus* and *Ae. albopictus*, respectively [15,16,17,18]. This aspect of pathogen-associated natural variation is beyond the scope of this review and is discussed in detail in [12,19].

The nature of variation in virus–host interactions is of great medical and evolutionary importance. Discerning general immune mechanisms and identifying determinants behind resistant individuals can inform the development of broadly acting intervention strategies to reduce arbovirus transmission and arboviral disease. Naturally segregating polymorphisms associated with resistance to viral infection can provide mechanistic insights into the control of viral infection, and identify loci likely under pathogen-mediated selection. These loci sometimes exhibit signatures of adaptive evolution or balancing selection, giving credence to the hypothesis of antagonistic coevolution between viruses and host immune genes. In this review, we provide a general description of antiviral immune mechanisms, and their impact across virus species studied in dipterans. We cover the nature and sources of variation in resistance among host individuals, particularly focusing on population studies of resistance polymorphisms and microbiota. For a list of viruses discussed in this review, see Table 1.

## 2. Antiviral Immune Processes

**Figure 1:**
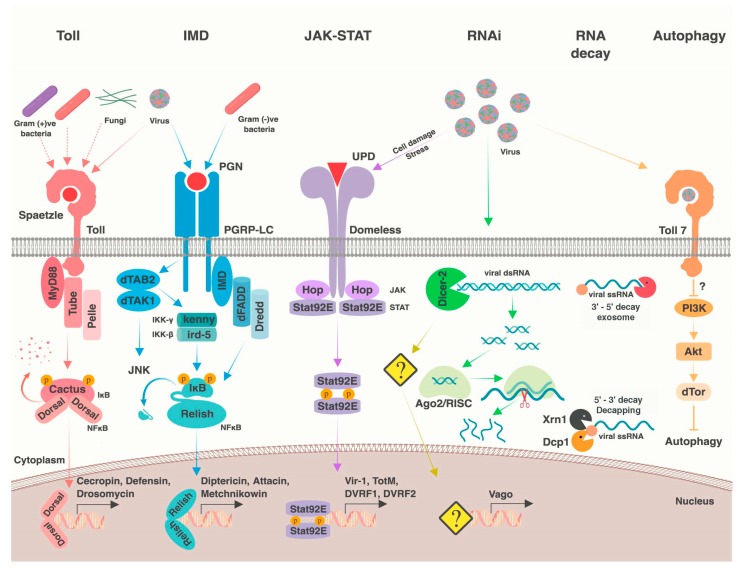
Antiviral innate immune pathways in dipteran insects. *Toll pathway*: Detection of pathogen-associated molecular patterns (PAMPs) by pathogen recognition-receptors leads to the proteolytic maturation of Spätzle, which binds to and activates Toll (dotted arrows). Activated Toll recruits adapter proteins MyD88, Tube and Pelle which targets Cactus for proteasomal degradation via phosphorylation. Cactus degradation releases the transcription factor Dorsal (Rel1 in mosquitoes) or Dif (Dorsal-related immune factor) when activated in response to bacterial infection. These translocate to the nucleus and activates the transcription of Toll pathway-regulated genes (e.g., *cecropin*, *defensin* and *drosomycin*). *IMD pathway*: Gram-negative PAMPs (e.g., peptidoglycan) bind to peptidoglycan recognition protein, PGRP-LC (or PGRP-LE) and signal through the adapter molecules IMD (Immune Deficiency) and dFADD (*Drosophila* Fas-associated death domain). This stimulates the caspase Dredd (Death-related ced-3/Nedd2-like protein), dTAK1 (*Drosophila* transforming growth factor β-activated kinase 1), and dTAK1 adapter protein dTAB2 (TAK1-binding protein 2). These proteins signal through the JNK pathway, and activate the IκB kinases kenny and ird-5, which phosphorylate the C-terminal tail of the transcription factor Relish (Rel2 in mosquitoes), leading to its subsequent activation via proteolytic cleavage by Dredd. Activated Relish translocates to the nucleus and drives the expression of genes regulated by the IMD-pathway (e.g., *diptericin*, *attacin* and *metchnikowin*). *JAK-STAT pathway*: Virus infection, possibly through induction of stress or cellular damage, triggers the activation of the JAK (Janus kinase)-STAT (signal transducers and activators of transcription) pathway, which begins with the binding of a cytokine of the unpaired (upd) family to the dimeric receptor, domeless. Subsequently, the receptor-associated JAK-tyrosine kinase hopscotch phosphorylates the cytoplasmic tail of domeless, leading to the recruitment of Stat92E. After Jak-mediated phosphorylation, Stat92E proteins dimerize and shuttle to the nucleus to activate the transcription of genes such as *vir-1*, *TotM*, *DVRF1* and *DVRF2*. *RNAi pathway*: Double-stranded RNA derived from virus replication intermediates are recognized and processed by Dicer-2 into 21 nt small-interfering RNAs (siRNAs), which are then loaded onto an Argonaute-2 (Ago2)-containing RNA-induced silencing complex (RISC). This complex degrades one of the two strands and uses the other strand as a guide RNA to target complementary viral sequences. Dicer-2 can also activate the expression of the cytokine *Vago* through an unknown pathway (visualized as ‘?’ in the figure). *RNA decay pathways*: Single-stranded viral mRNA can be targeted in the 3′–5′ direction through the RNA exosome degradation pathway. 5′–3′ degradation occurs through decapping enzymes Dcp1 and Dcp2 and the RNA exonuclease Xrn1. *Autophagy*: Some viruses can bind to the transmembrane receptor Toll-7, resulting in the induction of autophagy. This is most likely in an indirect manner by negatively regulating the PI3K (phosphatidylinositol 3-kinase)-Akt pathway.

Numerous conserved signaling pathways and cellular processes mediate antiviral immunity in dipterans (Figure 1) (reviewed in [1,2,4,5,6]). A subset of these pathways is generally antiviral (e.g., RNA interference), whereas others provide antiviral defense against only specific viruses (e.g., autophagy). In some cases, variation in viral resistance or tolerance can be mapped directly to genetic variation within these pathways. Although our focus is on dipteran immunity, we note that most of these processes are widely conserved, from flies to mammals. In this section, we briefly review known antiviral immune mechanisms in dipterans, which we will later discuss in a comparative context in Section 3.

### 2.1. RNA Interference

RNA silencing pathways utilizes short RNA sequences bound to an Argonaute-family protein to transcriptionally or post-transcriptionally silence complementary “target” sequences. These include the microRNA (miRNA) pathway, which primarily regulates endogenous gene expression, the PIWI-interacting RNA (piRNA) pathway, which regulates transposons, and the small interfering RNA pathway (siRNA), which serves as one of the most important antiviral defenses in invertebrates. Briefly, Dicer-2 (Dcr-2), an RNase III family endonuclease, recognizes viral-derived dsRNA and cuts it into 21 nucleotide siRNA duplexes. A single strand of the siRNA duplex is loaded into the Argonaute-2 (Ago2)-containing RNAi Induced Silencing Complex (RISC), which then slices any viral sequence that is complementary to the loaded siRNA, thereby controlling virus infection. In *Drosophila*, the siRNA pathway provides broad protection against both RNA and DNA viruses [20,21]. The siRNA pathway has been shown to be antiviral against arboviruses in relevant vector mosquitoes as well [22]. Recently, “secondary siRNAs” have been described, which are produced from reverse-transcribed viral DNA circles. These secondary siRNAs are excreted by hemocytes in exosomes and are proposed to mediate a non-cell autonomous, systemic RNAi-based immune response [23,24,25] (further discussed in Section 6.3).

In addition to viral siRNAs, *Aedes* mosquito species produce virus-derived piRNAs. This pathway is crucial for transposon suppression in the germline and does not have an antiviral role in *Drosophila* [26]. However, the PIWI genes have undergone expansion in mosquitoes and generate virus-derived piRNAs in the mosquito soma [27,28,29]. Depletion of viral piRNAs in mosquito cells by knocking down the associated proteins (Ago3 and Piwi5) seemed to have no effect on viral replication [30]. However, silencing Ago3 in *An. gambiae* mosquitoes led to increased O’nyong-nyong virus (ONNV) replication [31]. In addition, another piRNA pathway component, Piwi4 has been shown to be antiviral against Semliki forest virus (SFV) and Zika virus [32,33].

### 2.2. Other RNA Processes

In addition to RNAi, other RNA-based cellular processes have been implicated in antiviral defense. The RNA decay pathway protects against defective cytoplasmic RNAs, including those without 5′ caps or polyadenylated tails, lacking stop codons (nonstop decay), encoding early stop codons (nonsense-mediated decay), or stalled in ribosomes (no go decay) (reviewed in [34]). Offending RNA molecules are deadenylated and either degraded 3′ to 5′ by the RNA exosome, or decapped and degraded by the 5′ to 3′ exonuclease Xrn1 (reviewed in [35]). Viral RNA may include a number of hallmarks of aberrant cellular transcripts, including 5′ triphosphate groups, limited poly-A tails, or interior stop codons, making them susceptible to the RNA decay machinery [36,37]. Consistently, in *Drosophila*, the decapping enzymes, DCP1 and DCP2, and the RNA exosome have antiviral activity, and RISC-mediated silencing is partially dependent on Xrn1 and the exosome [38,39,40,41]. Additionally, there is evidence of viral modulation of processing body (P body) and stress granule formation. For example, Cricket paralysis virus (CrPV) infection leads to dispersal of P body components, which are sites that may be associated with organized decapping and 5′ to 3′ decay [42]. In mosquitoes, evidence for antiviral RNA decay comes from the flavivirus noncoding RNA, sfRNA, which is produced by stalling of Xrn1 on structured RNA elements in the 3′ UTR [43].

RNA editing by adenosine deaminases that act on RNA (ADAR) occurs during the mammalian innate immune response, where the replacement of adenosines with inosines reduces the stability of dsRNA and the accuracy of replication and translation, due to inosines being read as guanosines (reviewed in [44]). Evidence in support of an antiviral activity of ADAR in insects comes mainly from mutation and substitution patterns in a subset of RNA viruses. Two sequenced strains of sigma virus (DMelSV) appeared to have been hypermutated from A to G [45], and analysis of DMelSV variation within flies and across populations found that ADAR-resistant sites are less likely to be polymorphic [46]. Similar patterns are also observed in Zika virus, although it is unknown whether this is insect-mediated [47].

### 2.3. Nuclear Factor κB Pathways

There are two Nuclear factor κB (NF-κB) signaling cascades in flies: the Toll and immune deficiency (IMD) pathways (reviewed in [48,49]). Generally, these pathways are activated when an upstream pattern recognition receptor interacts with a pathogen-associated molecular pattern. This leads to phosphorylation of IκB (inhibitor of κB; encoded by *cactus* for the Toll signaling and by the C-terminal domain of *Relish* (*Rel2* in mosquitoes) in IMD signaling) by an IκB kinase (IKK, encoded by *pelle* in Toll signaling and *kenny* in IMD signaling). This results in degradation of IκB and subsequent release of the NF-κB transcription factor (encoded by *dorsal* (*Rel1* in mosquitoes) or *Dorsal-related immune factor* for Toll signaling and *Relish* for IMD signaling). These transcription factors then translocate into the nucleus to induce transcription of immune effectors, including antimicrobial peptides [48,49]. Activation of IMD may also signal through the JNK pathway [50,51]. Ostensibly, the primary functions of these pathways are in anti-bacterial and anti-fungal defense, although both have been implicated in defense against various dipteran viruses [21,52,53,54,55,56,57]. In agreement with an antiviral function, some insect viruses encode suppressors of the NF-κB pathway [58,59].

The antiviral effectors downstream of NF-κB are, for the most part, unknown, besides a couple of examples. The NF-κB-responsive antimicrobial peptides are slightly upregulated after viral challenge, and some seem to have antiviral properties [60,61]. However, the antiviral activity of the Toll and IMD pathways may be based on mechanisms independent from antimicrobial peptides. For example, Toll signaling seems to be involved in differentiation of hemocytes, phagocytic cells in the hemolymph of invertebrates (see Section 2.6 and Section 3.3 for the role of phagocytosis in immunity) [62]. IMD signaling can be pro-apoptotic, which itself can have antiviral functions [63,64], and is involved in *Pvf2* upregulation which activates antiviral ERK signaling [65,66].

### 2.4. JAK-STAT Pathway

In insects, the JAK-STAT pathway is activated upon binding of one of the unpaired ligands (upd, upd2, or upd3) to the pathway receptor, domeless. This interaction results in activation of the JAK kinase hopscotch, which then phosphorylates Stat92E, resulting in its dimerization and translocation into the nucleus where it induces transcription of JAK-STAT dependent genes (reviewed in [67]). The antiviral gene *Vago*, which is induced downstream of dsRNA recognition by Dcr-2, may also activate JAK-STAT signaling in mosquitoes, although possibly through a different receptor [21,68,69,70]. During viral infection, *Vago* and the *upd* ligands are upregulated, as well as a subset of known STAT-regulated genes [71,72]. This activation ostensibly results in an antiviral transcriptional program, and altering pathway activity in infected flies and mosquitoes affects resistance to some viruses [21,69,70,71,73]. Similar to antiviral NF-κB signaling, there are virus-specific differences in STAT-responsive transcriptional output, and STAT-responsive antiviral effectors are still mostly unknown. However, *attC* is upregulated downstream of JAK-STAT following Sindbis virus (SINV) infection in *Drosophila*, and heterozygous *attC* mutations lead to increased viral replication [61]. Additionally, two JAK-STAT pathway candidate effectors have been identified in Dengue virus (DENV)-infected *Ae. aegypti.* These DENV restriction factors (DVRF) include DVRF1 (CG15168 in *D. melanogaster*), a putative transmembrane protein which could serve as a receptor, and DVRF2 (CG8541 in *D. melanogaster*), a predicted secreted protein with anti-freezing and allergen domains [73]. Neither of the *Drosophila* DVRF homologs have been implicated in antiviral immunity.

### 2.5. Nutrient Signaling

Broadly speaking, diet and nutrient availability are intricately linked to immune variation and function (reviewed in [74,75]). Known diet-responsive signaling pathways have been implicated in antiviral immune modulation in dipterans, including ERK and PI3K/Akt signaling. Both ERK and PI3K/Akt are downstream of insulin receptor signaling, and PI3K/Akt has crosstalk with the amino acid-sensing TOR signaling pathway [65,76]. The ERK pathway can restrict viral infection in *Drosophila* midguts and mosquito cells [65,66]. In addition, downregulation of PI3K/Akt signaling can enhance antiviral defense, at least partially through releasing its inhibition of antiviral autophagy [77,78,79]. Akt also inhibits FOXO, a virus-induced transcription factor downstream of Toll, which upregulates antimicrobial peptides and possibly RNAi pathway components [80,81,82]. Notably, PI3K/Akt activity is proviral independent of its role in autophagy regulation, and is activated by SINV to promote cap-dependent translation [83].

### 2.6. Apoptosis, Phagocytosis, and Autophagy

Apoptosis, phagocytosis, and autophagy make important contributions to dipteran antiviral defense. Apoptosis is long known to be an important host defense response in vertebrates (reviewed in [84]). In *Drosophila*, viral infection induces p53-mediated transcription of RHG genes (*reaper, hid, grim*, and *sickle*), which promote degradation of Drosophila inhibitor of apoptosis 1 (DIAP1) and consequent activation of the initiator caspase Dronc [64,85]. This response occurs rapidly after infection, effectively reducing the duration a virus can access host factors that are crucial for replication [64]. Additionally, an N-terminal degron (a protein domain that regulates protein turnover rate) renders DIAP1 inherently unstable [86], allowing the promotion of apoptosis during virus-mediated translational inhibition [87]. Phosphatidylserine on apoptotic cells is recognized by the engulfment receptors draper and Integrin βν on phagocytes, which eliminate infected cells [88]. Individual viral particles, including Drosophila C Virus (DCV) and White spot syndrome virus (a natural shrimp nimavirus) may also be direct targets of phagocytosis in cell culture [89,90].

Autophagy, a process in which intracellular particles are enveloped by membrane crescents and shuttled to lysosomes for degradation, is also antiviral in some contexts [77,79,91]. For example, noncanonical Toll-7 signaling may be responsible for activating antiviral autophagy during rhabdovirus and bunyavirus infection, although autophagy is dispensable against most other viruses [56,78,79].

## 3. Virus-Specific Responses

Virus–host interactions include both general and virus-specific components. For example, a genome-wide RNAi screen of West Nile virus (WNV) uncovered 50 restriction factors, over half of which were antiviral across flaviviruses, whereas only seven were broadly antiviral across disparate viral families [92]. This indicates that hosts have evolved antiviral mechanisms in response to virus-specific infection cues (e.g., pathogen associated molecular patterns, pathogenesis, or affected tissues), and/or that viruses are able to evade a subset of the host immune responses. Comparative antiviral immunity experiments have primarily focused on RNAi, induced immune pathways and their downstream transcriptional responses (i.e., NF-κB and JAK-STAT pathways), and cellular responses. These studies have used a collection of native and non-native viruses (Table 1) and shown considerable variation in the efficacy of cellular and humoral immunity across viruses, concomitant with highly virus-specific transcriptional responses to infection [6]. Notably, there has been recent interest in identifying and isolating native dipteran viruses, focusing both on viruses from diverse families and those with nearby relatives of medical importance, representing a clear potential for dipterans to become a powerful in vivo comparative system to study the diversity of antiviral immune responses [93,94,95,96].

### 3.1. RNA Interference

The RNA interference pathway is often referred to as being the most general and important innate antiviral immune pathway in insects. Indeed, RNAi is demonstrably antiviral against a spectrum of RNA and DNA viruses across insects [3,22]. The broad importance of antiviral RNAi is underlined by the prevalence of viral suppressors of RNAi (VSRs), often found in insect-infecting viruses, which inhibit processing of viral dsRNA by RNAi enzymes [97,98,99,100,101,102]. These VSRs may vary in potency, host-specificity, and mechanism, although commonly act by binding either short or long dsRNA to shield it from Dcr-2 or Ago2 processing [102,103]. In some cases, a VSR may block RNAi function so completely that removing key RNAi components appears not to have an effect on virus replication, and thus variation in VSR efficacy could lead to observed virus-specific variation in the requirement of antiviral RNAi. For example, Nora virus VP1 encodes an Ago2 suppressor, implying that RNAi poses an antiviral threat to Nora virus [20]. However, Nora virus titers remain unchanged in *Dcr-2*, *r2d2*, and *Ago2* mutants during persistent infection [104].

VSR potency may also be reflected in length distributions of virus-derived small RNAs. Canonically, virus-derived small RNAs are overwhelmingly 21 nucleotides in length, because Dcr-2 cleaves at 21 base intervals. However, a metagenomic survey of viruses in *D. melanogaster* found variation in virus-mapping small RNA length distributions [94]. Four *D. melanogaster*-infecting picorna-like viruses (DCV, Nora virus, Kilifi virus, and Thika virus) have broad length distributions, with similar amounts of 22–29 nucleotide viral-derived small RNAs as 21 nucleotide species. Because two of the four viruses with broad siRNA length distributions are known to suppress RNAi, a viable hypothesis is that especially potent VSRs may reduce the number of Dcr-2-generated siRNAs, although increased viral degradation products and the concomitant increase in 22–29 nucleotide species could also explain the pattern [94]. Regardless, the prevalence of VSRs underlines the importance of considering virus-mediated subversion of host immunity when studying virus-specific responses.

### 3.2. Induced Immune Responses

Viruses trigger a rapid transcriptional response in infected flies [105]. This depends on recognition of virus-associated molecular patterns, and on other infection-derived cues such as host manipulation or damage (reviewed in [4]). As in mammals, NF-κB and JAK-STAT pathways help coordinate these inducible responses, and a subset of upstream signaling pathway regulators are often differentially expressed during infection with different viruses [21,71,72,106,107]. In *Drosophila*, the Toll and Imd pathways are antiviral against a broad panel of RNA viruses. Toll signaling is antiviral upon oral, but not systemic, viral infection, and mutants are less resistant to DCV, CrPV, Nora virus, Drosophila X virus (DXV), and Flock House virus (FHV) [52,57]. Likewise, IMD has been proposed to be antiviral against DCV, SINV, and CrPV [54,55,66]. The antiviral role of NF-κB pathways in mosquitoes is not as apparently widespread. The Toll pathway was found to have an antiviral role only against the flavivirus DENV in *Ae. aegypti* mosquitoes [53,108], whereas no role of this pathway was observed for the alphaviruses SFV and ONNV [109,110]. On the other hand, the IMD pathway was found to be effective against SFV and ONNV when stimulated prior to infection in cell culture [109,111]. Even though some IMD pathway components and effector genes were found to be upregulated during DENV infection, transient activation of the pathway did not affect viral titers [53].

The antiviral role of JAK-STAT signaling has also been investigated across *Drosophila* viruses, where it is crucial in CrPV and DCV infections, but exerts a minimal effect on FHV, SINV and vesicular stomatitis virus (VSV) [21,71]. However, JAK-STAT controls replication of a SINV replicon, indicating there may be JAK-STAT-dependent responses specific to tissues or the route of infection [54,112]. In mosquitoes, the antiviral effect of the JAK-STAT pathway has been seen in DENV-infected *Ae. aegypti* mosquitoes, where silencing components of the pathway resulted in higher viral titers and activating the pathway had the opposite effect [70,73]. Similar to the IMD pathway, stimulating the JAK-STAT pathway prior to infection had an antiviral effect on SFV [109]. However, the antiviral role of this pathway in other virus–vector combinations remains unclear, where no upregulation of JAK-STAT pathway components was observed upon infection (reviewed in [113]).

The resulting downstream transcriptional response across viruses is remarkably variable [4,21]. Comparison of the differentially expressed genes following infection with DCV, SINV, or FHV resulted in the identification of 601 genes, only 42 of which are shared between the three viruses [21]. An exemplar of this trend is an analysis of JAK-STAT responsive genes *vir-1* and *TotM*, which show an almost mutually exclusive expression pattern in response to a panel of viruses, whereby *vir-1* is strongly induced in response to CrPV, DCV, and FHV, while *TotM* is induced after SINV, VSV, or DXV infection [21]. Neither gene is strongly upregulated in response to the DNA viruses IIV6 or Kallithea virus [21,107]. Likewise, the heatshock pathway is induced in vivo in response to DCV and CrPV, but not IIV6 [72]. *Diedel*, a negative regulator of Imd signaling, also has highly virus-specific regulation, and is induced over 100-fold during SINV and VSV infection, but less than 5-fold in response to CrPV, DCV, and FHV [59]. However, there may be responses common to a particular virus family or even between viruses of different families. A comparative transcriptomic analysis of infections by the flaviviruses DENV, Yellow fever virus (YFV) and WNV infection in *Ae. aegypti* mosquitoes found 35 genes that were commonly differentially expressed, suggesting a transcriptomic signature unique to flaviviruses. Antimicrobial effectors of the JAK-STAT pathway (four Cecropin A-like genes and one defensin gene) as well as components of the Toll pathway were found to be downregulated by all three viruses [114]. A more recent study compared transcriptional responses between flaviviruses, alphaviruses and bunyaviruses upon infection of *Ae. aegypti* mosquitoes. Only 19 genes were found to be upregulated by all tested viruses and no commonly downregulated genes were found. Among the 19, the gene responsible for GABA signaling was found to be connected to blood feeding and responsible for enhancing virus replication [115]. Together, these studies indicate the transcriptional response to a particular virus is unique, and may be the amalgamation of host adaptation to a specific virus, virus-mediated damage and immune subversion, cellular and tissue tropism, and virus replication kinetics.

### 3.3. Autophagy, Phagocytosis, and Apoptosis

A recent study using *Drosophila* has formally compared the requirement of phagocytosis, apoptosis, and autophagy using a panel of six viruses (DCV, CrPV, FHV, VSV, SINV, and IIV6) [79]. Interference with phagocytosis by latex bead injection or genetic ablation of hemocytes led to increased susceptibility to CrPV, FHV, and VSV, but not to IIV6, DCV, or SINV [79]. Antiviral phagocytosis likely operates through different mechanisms. CrPV and FHV induce apoptosis, and phagocytes were recruited to dying cells to remove them [79,116]. However, VSV did not induce apoptosis, indicating the effect from phagocytes may be through direct clearance of viral particles, or, more speculatively, via the recently described secondary siRNA pathway [25] (described in more detail in Section 6.3). Additionally, DCV infection induced apoptosis and subsequent phagocytic clearance, although phagocytosis-deficient flies were not more susceptible to infection [79,88]. Finally, this study found that autophagy-deficient flies were more susceptible to VSV [77], but not to DCV, CrPV, FHV, and IIV6 infections [79]. Additionally, autophagy appears to be proviral in FHV infection, where flies mutant for *Atg7*, a gene required for autophagosome formation, had lower FHV titer and less virus-induced mortality [79].

## 4. Genetic Variation in Antiviral Immunity in Dipterans

The evolutionary relationship between host and pathogens is often referred to as antagonistic coevolution, because an increase in the fitness of one often corresponds to a decrease in the fitness of the other. Framing host–parasite interactions in an evolutionary context is critical, as variation in epidemiological and population genetic parameters (e.g., host costs of resistance, demographic population structure, and pathogen virulence and transmissibility) is expected to result in different types of selection, which in turn affects the architecture of genetic variation in resistance [117]. Even with relatively simple genetic architectures, where resistance and susceptibility are controlled by few host and parasite genotypes at single loci, antagonistic coevolution can result in a resistant allele being maintained at a stable frequency, changing in frequency chaotically or cyclically, or fixing in a population (e.g., [117,118,119]). In reality, host–parasite interactions can be immensely complex [120], and most of the loci under pathogen-mediated selection are unknown. Identification of these loci is of great medical and evolutionary importance, and efforts have been made to characterize genetic variation in host resistance, and to determine the effects of parasite mediated selection on host gene evolution.

### 4.1. Segregating Genetic Variants Associated with Viral Resistance in *Drosophila*

In some cases, genetic variation has been mapped to discrete loci in the host genome. In *D. melanogaster*, the most extensively studied host–virus system at the population level is DmelSV, a negative-sense ssRNA rhabdovirus (reviewed in [121]). DmelSV is transmitted vertically to offspring through eggs and sperm, although some fly strains are not permissive to DmelSV replication [122]. This resistance was first mapped to a complex amino acid substitution in the N-terminal PB1 domain of *ref(2)P* (Table 2), a gene now known to be involved in autophagy and Toll signaling [122,123,124,125,126]. Viral replication and transmission is reduced in homozygous *ref(2)P* mutants or trans-heterozygotes bearing a mutant *ref(2)P* and the refractory allele, indicating that ref(2)P is a proviral host factor in DmelSV infection [127].

More recently, two additional loci have been mapped with segregating variants associated with resistance to DmelSV. The first is a triallelic polymorphism at the *CHKov1* and *CHKov2* paralogs [131], where an insertion of a transposable element (*Doc* element) in *CHKov1* provides resistance to DmelSV, and a following complex (and rare) rearrangement is associated with even greater DmelSV resistance [131] (Table 2). The Doc insertion also confers resistance to pesticides [139]. Although the functional relevance of the *CHKov* genes during viral infection remains untested, the resistance mutations could exert an effect on viral entry, as *CHKov* is a predicted acetylcholine esterase, and Rabies virus (also a rhabdovirus) uses the acetylcholine receptor as a cell entry point [140]. In addition to *ref(2)P* and *CHKov*, a large deletion in the serine-rich linker region of *Ge-1* results in a 10-fold reduction in viral titer and lower infection rates (Table 2) [40,128]. Ge-1 is a conserved adaptor bridge between Decapping protein 1 and Decapping protein 2 that helps localize these enzymes to processing bodies [141,142], and thus could plausibly exert its antiviral effect through the RNA decay pathway [40]. Finally, up to 7 QTLs associated with DmelSV replication or transmission have been identified in genetic mapping experiments, however the exact loci responsible have yet to be reported [128,132,143,144].

Natural variation has also been mapped for two other *Drosophila* viruses: DCV and Kallithea virus. Resistance to DCV has been mapped to complex polymorphisms at the *pastrel* (*pst*) locus [130,133]. The primary *pst* resistance mutation is due to a single amino acid change, although there are multiple structural alleles and *cis-*regulatory changes that may enhance resistance, resulting in seven alleles with four distinct phenotypes [134]. Although the function of *pst* is unknown, overexpression of the susceptible allele provides protection against DCV, and *pst* is upregulated after intra-abdominal viral injection, indicating that *pst* is an induced antiviral factor [134]. In addition to *pst,* polymorphism in Anaphase promoting complex 7, *Ubiquitin conjugating enzyme E2H,* and 2 QTLs may also underlie genetic variation in DCV resistance, although these await more extensive characterization [130,133].

A genome-wide association study has found multiple loci that are associated with either viral titer or mortality following Kallithea virus infection, a dsDNA nudivirus of *D. melanogaster* [94,107]. The most confident association was a nonsynonymous polymorphism affecting a subset of splice variants in *Cdc42-interacting protein 4* (*Cip4*), a gene involved in membrane trafficking [107,145]. Many other loci were found significantly associated to Kallithea virus resistance (>50 genes), in stark contrast to the above RNA viruses, where variation in resistance is explained by few large effect loci. This could reflect general differences in coevolution between hosts and their DNA and RNA viruses, such as the greater complexity (Kallithea has approximately 100 genes) and reduced substitution rate of DNA viruses [146].

### 4.2. Evolution of Resistance Loci

The genetic architecture of resistance to viruses in *Drosophila* often seems to include large-effect polymorphisms at an intermediate frequency in populations (Table 2). These variants are likely under pathogen-mediated selection, and surrounding patterns of polymorphism and divergence have been compared to expected patterns of balancing selection (i.e., selection that maintains genetic variation) or recurrent positive selection (i.e., selection on an advantageous allele), which would be compatible with their involvement in antagonistic coevolution (reviewed in [117,147,148]). Although none of the identified resistance loci display significantly increased diversity that is a hallmark of balancing selection, *ref(2)P* and *pastrel* have relatively high levels of nonsynonymous or structural polymorphism [123,130,134,149]. This may be due, in part, to incomplete selective sweeps, whereby a resistance mutation rises to high frequency and subsequently loses the selective benefit through viral counter-adaptation. This was witnessed in the 1980s, when a DmelSV strain that regained transmission advantage in flies with a resistant *ref(2)P* allele swept through the population [150,151,152,153,154]. Additionally, some dipteran immune genes exhibit unusually high diversity. NF-κB-responsive AMPs appear to be under balancing selection and show high levels of nonsynonymous polymorphism, with some convergently maintained in different species, likely due to their role in anti-bacterial or anti-fungal immunity [155,156]. The N-terminal glutamine-rich repeat region of *Ago2* is also hypervariable either due to diversifying selection or high mutation rates and low constraint [157,158].

In addition to maintaining polymorphism, pathogen-mediated arms races may be expected to fix adaptive mutations. This can occur quickly on an evolutionary timescale, and these beneficial mutations will only be briefly visible as variation within populations. However, they can be recognized as elevated divergence between populations or species. This is evident in genes with segregating resistance polymorphism, and *ref(2)P*, *CHKov1/2*, and *Ge-1* show signs of recent or recurrent positive selection [40,131,153]. These patterns are also apparent in some immune genes, and are most striking in the RNAi pathway, which evolves rapidly due to adaptive evolution, particularly in genes mediating defense against transposons and viruses, including antiviral effectors *Dcr-2* and *Ago2* [159,160,161]. In addition, RNAi genes are more likely to show diversity patterns consistent with positive selection driving new mutations to fixation [161,162,163,164]. Although less likely to be virus-mediated selection, genes in the Toll and IMD signaling pathways may also have elevated levels of positive selection, with the most convincing evidence in *Relish* and its interactors [165,166,167]. Additionally, genes encoding pathogen recognition proteins appear to evolve rapidly, especially those involved in phagocytosis [166,168].

### 4.3. Genetic Variation Associated with Viral Resistance in Mosquitoes

Although the genomic tools of mosquitoes are not as advanced as those for *Drosophila*, the sequencing of the genome of *Ae. aegypti* (the principal vector of Dengue, Chikungunya and Zika viruses) [169] has enabled the investigation of the precise genetic determinants of vector competence. Prior to the genomics age, *Ae. aegypti* strains with differences in midgut and disseminated DENV infections were identified [170,171] and used to map alleles associated with susceptible or refractory phenotypes. Quantitative-trait loci (QTL) mapping allowed the determination of several loci that act additively to determine midgut infection and dissemination [137,171,172]. Two QTLs were identified that significantly associated with DENV-2 midgut infection [137]. One of these contained *early trypsin*, a female-specific gene induced shortly after blood-feeding, and required for proteolytic digestion of the bloodmeal [173]. A subsequent fine-scale mapping study of the segregating sites in the *early trypsin* coding sequence in four Mexican populations was unable to find a causal variant, indicating that the locus may be linked to *early trypsin* in the original QTL, or that polymorphic *cis*-acting elements are causing differences in *early trypsin* expression [174]. Regardless, there is evidence that trypsins are important regulators of DENV infection, framing this QTL in an interesting context. For example, addition of a soybean trypsin inhibitor to an infectious blood meal significantly reduced DENV midgut infection and dissemination [175]. In contrast, another group showed that adding soybean trypsin inhibitor as well as RNAi knock-down of the late trypsin gene *5G1* led to higher DENV infectivity of *Ae. aegypti* mosquitoes [176]. An expressed sequence tag (EST) with high homology to a trypsin inhibitor was found in refractory *Ae. aegypti* populations, implying further that midgut proteolytic activity could limit DENV infection [177].

Another QTL mapping study found four genomic regions associated with variation in body size that may additionally affect vector competence of *Ochlerotatus triseriatus* mosquitoes for La Crosse encephalitis virus (LACV) [178]. This virus belonging to the California serogroup of bunyaviruses is actively maintained by transovarial transmission from infected females to their progeny in the intervening periods between epizootic outbreaks, during which the virus maintains low level replication that is not detrimental to the overwintering embryos [179]. Three genetic loci were found to additively determine transovarial transmission rates in *Ocherotatus triseriatus* mosquitoes for LACV (Table 2) [138].

Genetic differentiation among various *Ae. aegypti* strains from Vietnam and Thailand, possibly caused by insecticide use, were also found to be associated with vector competence for DENV [180,181]. Lambrechts and colleagues determined that there is an active interaction and ongoing local adaptation between vector and virus genotypes, resulting in genotype-by-genotype (GxG) interactions that affect vector competence phenotypes [9]. Population genetic studies to map the mosquito loci underlying these GxG interactions identified QTLs that conferred resistance to several DENV viral strains and others where a specific QTL varied based on the DENV isolate or serotype used [136]. Further fine-scale mapping of these GxG interactions revealed that polymorphisms in the *Dcr-2* locus correlated with DENV susceptibility in an isolate specific manner. Thus, variation in dsRNA binding or cleavage by Dcr-2, coupled with variation in the relative importance of antiviral RNAi across DENV strains could explain the observed G×G interactions, although the precise mechanism remains to be characterized [135]. As in *Drosophila*, RNAi genes in *Aedes* may have higher rates of protein evolution [182]. Mosquitoes do not have an overtly antagonistic relationship with the arboviruses they transmit, leading to the speculation that it is not arboviruses, but insect-specific viruses [183] or transposon movement that could drive such evolution.

Lately, transcriptomic differences between refractory and susceptible vector populations have been used to identify causal determinants of variation in vector competence, with most studies focusing on *Ae. aegypti* and DENV. Behura et al. identified different gene networks being activated in susceptible versus refractory populations [184]. In particular, apoptosis-related genes were expressed at higher levels in refractory populations, which may be a defense response that impedes a productive DENV infection. For example, a mosquito ortholog of the Drosophila pro-apoptotic gene *reaper*, termed *michelob_x*, as well as other related genes, were upregulated upon DENV infection only in a refractory *Ae. aegypti* strain [64,177]. In agreement, knock-down of the initiator apoptotic caspase *AeDronc* in a refractory *Ae. aegypti* strain increased its permissiveness to DENV infection, whereas suppressing the caspase inhibitor *AeIAP1* in susceptible strains made them refractory [185]. Differential regulation of apoptosis in mosquitoes with a refractory phenotype has been documented in other vector-virus combinations as well [5,186]. For example, WNV infection showed apoptotic effects in the midguts of a refractory population of *Cx. pipiens* mosquitoes, and clearance of WNV infection in the salivary glands over time correlated with levels of apoptosis in *Cx. quinquefasciatus* [187,188].

In addition to apoptosis-related factors, expression of furin-like genes were higher in the susceptible strains, suggesting that they may aid in DENV maturation [184]. Expression of many cuticle protein genes were comparatively lower in the susceptible population, suggesting that they may possess thinner anatomical barriers that enhance midgut infection and escape [184]. A putative 67 kDa DENV receptor protein (R67/R64) on *Ae. aegypti* midgut epithelial cells was found to be expressed at significantly higher levels in the midguts of strains susceptible to DENV infection as compared to refractory ones [189]. Interestingly, a glucosyl/glucuronosyl transferase (*AAEL003099*) was found to be downregulated in *Ae. aegypti* populations refractory to DENV [190] and the same gene was found to be upregulated by a *Talaromyces* species of fungus that increases the mosquito’s susceptibility to DENV [191]. These studies suggest that the relative expression levels of these genes in mosquito populations could be a source of natural variation that influences vector competence.

Several genes belonging to innate immune pathways (Toll, JAK/STAT, IMD) were selectively upregulated in refractory *Ae. aegypti* strains, suggesting that the basal expression level of immune response genes may modulate susceptibility to DENV infection [190]. The same study also highlighted the importance of differential vATPase subunit expression in influencing vector competence. These multi-subunit enzymes drive proton transport to acidify organelles like endosomes and lysosomes [192] and is required during a key step in the DENV life cycle, low pH-mediated viral and cellular membrane fusion [193]. Indeed, RNAi-mediated knock-down of the vATPase G subunit gene (*AAEL012819*) rendered susceptible strains more refractory to DENV [190].

## 5. Microbe-Dependent Variation in Dipteran Antiviral Immunity

In addition to genetic determinants in the host genome, the fly microbial community may influence resistance and tolerance to some viruses, where inter-strain variation in antiviral protection can manifest itself as variation among fly individuals. The effect of *Wolbachia* infection has been the primary focus of these studies, although the importance of the gut microbiome is beginning to be understood.

### 5.1. *Wolbachia* in *Drosophila*

*Wolbachia* is a α-proteobacterium widespread among insects that infects reproductive tissues and induces a diverse array of phenotypes across insect hosts, including male-killing, male feminization, cytoplasmic incompatibility, and increased resistance to RNA viruses [194,195,196]. The exact mechanism behind *Wolbachia*-mediated antiviral protection has remained elusive. *Wolbachia* titer is generally correlated with the magnitude of antiviral protection [197,198,199,200], which has been explained by competition between viruses and *Wolbachia* for resources or by *Wolbachia*-mediated immune priming [201]. A cholesterol-rich diet ameliorates *Wolbachia* pathogen interference against DCV in *Drosophila*, but altering nutrient availability has no effect on DENV in mosquitoes [202,203]. Although Ago2, reactive oxygen species, certain AMPs, and Mt2 (a methyltransferase with antiviral or proviral activity in different contexts) are differentially regulated during *Wolbachia* infection [204,205,206,207,208], the effects are small, or species specific, making it unlikely that immune priming is the sole mechanism of viral interference [207,209,210,211,212]. Regardless of mechanism, different *Wolbachia* strains have different propensities toward pathogen interference [200]. Natural variation in antiviral protection of *Wolbachia* has been investigated in *D. melanogaster* and *D. simulans*, where *Wolbachia* infection is generally protective against RNA (but not DNA) viruses [195,199].

Three genotypes of the single *Wolbachia pipientis* strain that infects *D. melanogaster* have been extensively characterized for antiviral protection. The most prevalent genotype in the wild, *w*Mel, provides limited antiviral protection, whereas the other two, *w*MelCS and *w*MelPop, provide intermediate and strong antiviral protection, respectively [213]. Antiviral protection in these strains is positively correlated with *Wolbachia* titer and virulence, with *w*MelPop causing considerable mortality in infected flies [213,214]. The extreme phenotypes of *w*MelPop have been mapped to a copy number variant encompassing eight *Wolbachia* genes, called Octomom [213,215,216]. Octomom copy number varies within and across flies infected with *w*MelPop, where higher copy number *w*MelPop genotypes replicate more rapidly, have a higher virulence, and provide greater protection against RNA viruses [213,216,217]. *Wolbachia*-mediated antiviral protection is likely more complicated than this single locus, as *w*MelCS and *w*Mel3562 (another highly virulent *Wolbachia* strain) have single or low Octomom copy numbers [213,218], and *Wolbachia* with Octomom deletions retains its pathogenicity (although this genotype has not been assayed for pathogen interference) [215,219]. Notably, the *w*MelPop strain was recovered from laboratory flies, and has not been found in the wild [213,214].

Many *Wolbachia* strains are able to subvert host reproduction, benefiting infected females and driving the infection through a population [220]. Because antiviral protection and reproductive subversion appears to be uncorrelated [221], this could lead to waves of viral resistance and susceptibility in fly populations. For example, comparison of *Wolbachia* and mitochondrial sequences indicates that the *w*Mel strain has recently swept to high frequency at the expense of the *w*MelCS strain, suggesting that worldwide populations of *D. melanogaster* may have recently become more susceptible to viral infection [222,223,224]. In addition, one of the four native *Wolbachia* strains that infect *Drosophila simulans* (of which two exhibit pathogen interference) has rapidly (within approximately 10 years) swept through populations in California and Australia [197,225,226,227]. This strain, *w*Ri, exhibits pathogen interference, suggesting that these *D. simulans* populations may have recently become more resistant to RNA viruses. As an estimated 40% of insect species may host *Wolbachia* and approximately half of *Drosophila*-infecting *Wolbachia* strains provide antiviral resistance [199], *Wolbachia* is likely to be a dynamic and sizable source of variation for virus susceptibility among individuals and species.

### 5.2. *Wolbachia* in Mosquitoes

*Wolbachia* is not found to be naturally associated with *Ae. aegypti*, although an *Ae. albopictus* strain from La Réunion Island naturally harbors two different strains of *Wolbachia* (*w*AlbA and *w*AlbB) [228]. These bacteria could reduce DENV transmission by limiting the amount of infectious virus particles secreted into the saliva, perhaps contributing to the reduced vector competence in this species relative to *Ae. aegypti* [228]. Artificial introduction of the *Wolbachia wMel* strain into *Ae. aegypti* limits DENV and CHIKV infection without adversely affecting mosquito life span [229]. This may allow the bacterium to spread and stably establish in the population, making it a promising candidate for introgression into *Ae. aegypti* [230]. Since 2011, there have been several efforts to release *Wolbachia*-infected *Ae. aegypti* into wild mosquito populations in Australia [231,232], showing the successful establishment of *Wolbachia* in *Aedes* populations and reduction of DENV transmission potential in field conditions [233]. Recently, the Eliminate Dengue Program, now known as the World Mosquito Program (https://www.worldmosquitoprogram.org), in partnership with organizations and governments of countries with high arbovirus transmission has initiated trials to evaluate the potential of *w*Mel infected *Ae. aegypti* to suppress arbovirus transmission. As it constitutes an artificial intervention, variation in antiviral immunity introduced by transinfecting *Ae. aegypti* with *Wolbachia* will not be discussed further in this review.

### 5.3. Gut Microbiota in *Drosophila*

The interplay between gut commensals and viruses has been best studied in mammals, where the microbiota can be pro- or anti-viral, dependent on the virus [234]. This appears to be true in insects as well. For example, baculoviruses reach higher titers and are more virulent in the moth *Spodoptera exigua* when the microbiota is present [235]. Conversely, in *D. melanogaster*, the microbiota signals through Relish to upregulate *Pvf2*, which in turn activates antiviral ERK signaling in the gut [65,66]. Antibiotic treatment leads to increased replication rates of DCV and VSV [66], although this does not affect virus-induced mortality [57]. Upregulation of *Pvf2* was strictly mediated by gram-negative bacteria (*E. coli* or *Acetobacter species)*, although some bacterial species may be more potent activators [66]. Older flies have an altered microbiota and concomitantly higher levels of reactive oxygen species, which renders them unable to upregulate *Pvf2* [236]. These phenotypes can be reversed by a faecal transplant from young to old flies, or with experimental association of old flies with *Lactobacillus* *fructivorans* and heat-killed *Acetobacter pomorum* [236]. The microbiota also influences gut environmental factors that likely have a role in antiviral immunity, including gut renewal rate and basal levels of JAK-STAT and IMD signaling [237]. Thus, it seems probable that the community structure of commensals plays an integral role in shaping the gut environment that enteric viruses encounter, and that variation in gut bacterial strains may translate into an important source of variation in natural populations. Indeed, microbiota is variable in wild flies [238,239]; however, frequencies of strains and species across worldwide populations is unknown, and little has been done to link specific commensals to antiviral resistance in flies [66,236].

### 5.4. Gut Microbiota in Mosquitoes

The mosquito midgut flora affects several mosquito traits, including development, nutrition, reproductive capacity and vector competence. Mosquitoes start acquiring their gut flora at the larval stage, the composition of which will be defined by the microbial flora of the larval aquatic habitat. The gut microflora is not static and changes later in the adult stage based on its sex, the flora acquired from nectar feeding, gut environmental changes through blood meals, and sometimes from venereal transmission (reviewed in [240]). As in *Drosophila*, the microbiota in mosquitoes may have complex and virus-specific effects on immunity and vector competence. The presence of different Gram-negative bacterial species such as *Proteus*, *Paenibacillus* and *Chromobacterium* in the *Ae. aegypti* midgut protects against DENV infection, whereas an opposite effect was seen for ONNV in its vector, *Anopheles gambiae*, in which treatment with antibiotics decreased susceptibility to ONNV [111,241,242]. Oral co-infection of a naturally occurring midgut bacterium of *Ae. aegypti*, *Serratia odorifera,* along with DENV or CHIKV increased its susceptibility to both arboviruses, presumably through the interaction of bacterial and mosquito midgut proteins [243,244]. In turn, the microbial community structure in the midguts of *Ochlerotatus triseriatus* and *Aedes japonicus* mosquitoes were significantly changed after a LACV infectious blood meal, suggesting an interaction between the vector gut microbiota and the infecting arbovirus, possibly influencing vector competence phenotypes [245].

These microbes could influence vector competence through different mechanisms, including innate immune priming and secretion of antiviral effectors. For example, the microbiota upregulates genes encoding antimicrobial peptides such as cecropins, defensins and lysozyme C that have known antiviral activity against DENV [53,60,241,242]. In addition, certain bacterial isolates belonging to the *Enterobacteriaceae* family, isolated from the midgut of *Ae. albopictus* were found to suppress replication of LACV in vertebrate cells, and a bacteria-free biofilm from *Chromobacterium* (isolate *Csp_P*) could inhibit DENV replication in vertebrate cells [242,246]. More recently, *Talaromyces* species of fungus isolated from the midgut of field-caught *Ae. aegypti* was shown to increase the permissiveness of the mosquito to DENV by secreting a hitherto unknown factor that downregulates trypsin gene expression and enzymatic activity in the gut [191]. This is line with the observation that a trypsin inhibitor or genetic knock-down of a late trypsin gene increased susceptibility to DENV [176]. Thus, the gut microbial flora is likely to be a dynamic source of natural variation for susceptibility to arboviral infection.

## 6. Other Sources of Variation

The resistance of an individual host is dependent on several other factors besides host genotype and the associated microbiota. The abiotic environment, sexual dimorphism, mating, infection history and endogenous viral elements have each been identified to influence infection outcomes. However, most of these factors have received disproportionate attention in either fruit flies or mosquitoes, and there have been few comparative studies of these effects across viruses, making generalizations between hosts and among viruses difficult.

### 6.1. Abiotic Environment

Variation in the abiotic environment, such as temperature, time of infection, or nutrient availability, can alter infection outcomes, either by interacting with the host immune system or the pathogen life cycle [74]. These factors have been investigated in laboratory experiments in *Drosophila*, although the ecological importance of each is not directly clear, as wild *Drosophila* have not been analyzed. For example, temperature influences the virulence of DmelSV and CrPV, and the titer of DmelSV and Kallithea virus in *D. melanogaster* [55,107,143]. Infection success may also depend on the time of day during which an infection occurs, as the antibacterial immune response appears to be more potent at night, in part due to a circadian rhythm-mediated increase in phagocytosis, although this has not been investigated in the context of viral infection [247,248,249]. Nutrient signaling also has substantial crosstalk with antiviral defense pathways, and starvation and macronutrient availability are known modulators of innate immunity [80,81,82,250,251]. Although associations between macronutrient profiles and efficacy of antiviral defense has not been assessed in *Drosophila*, African armyworms “self-medicate” with high protein, low carbohydrate diets after baculovirus infection and a similar diet leads to greater antibacterial defense in *D. melanogaster* [251,252]. Additionally, there is substantial genotype-by-nutrition variation in antibacterial immunity, and polymorphisms have been mapped which resistance to *Providencia rettgeri* in a diet-dependent manner [253]. Interestingly, most of these variants are in genes without a known role in immunity [253].

The interaction between immunity and the abiotic environment has been much better studied in mosquitoes, where vector competence is known to be modulated by variables emerging from different mosquito habitats. For example, larval environmental stressors like starvation, competition, elevated rearing temperatures, and low dose insecticide exposure were shown to increase the susceptibility of *Ae. aegypti* to SINV infection. This observation was attributed to the higher expression of antimicrobial peptides like *cecropin* and *defensin* as well as different stress-response genes like *HSP70*, *HSP83*, *transferrin* and *CYP6Z6* [254].

Reduced nutrient availability or larval overcrowding may lead to stress, starvation, and a smaller adult size in mosquitoes, although the ultimate effect of these variables on vector competence is not clear. Nutritionally deprived and smaller-sized *Ochlerotatus triseriatus* females were better at transmitting LACV due to the thinner basal lamina layer of their midguts, thereby allowing more viruses to disseminate into the hemocoel and reach the salivary glands and be subsequently transmitted [255]. Similarly, laboratory-derived smaller *Ae. aegypti* mosquitoes were more susceptible to DENV infection, resulting in higher dissemination rates (frequency at which an arbovirus can infect and replicate in the midgut epithelial cells and cross over into the hemocoel) ([256], but see [257]). In contrast, a recent vector competence study of LACV in field collected *Ochlerotatus triseriatus* mosquitoes showed the opposite result, where infected females were larger than uninfected ones [258]. Increased larval competition also resulted in increased DENV infection rates in *Ae. albopictus*, although no such effects were observed with the lab-adapted Rockefeller strain of *Ae. aegypti* [259]. However, in a recent investigation, smaller-sized larvae from a Trinidadian strain of *Ae. aegypti* exhibited reduced susceptibility to DENV [260].

For many virus–vector combinations, it has been observed that mosquitoes held at lower temperatures during the extrinsic incubation period (EIP, time period between the acquisition of a virus through an infectious blood meal to its subsequent transmission to a susceptible vertebrate host) show reduced infection and dissemination and/or transmission rates [261,262,263,264]. A higher temperature could lead to a shorter extrinsic incubation period which in turn could lead to an increased transmission potential, as has been reported in DENV-infected *Ae. aegypti* and *Ae. albopictus* mosquitoes [265,266]. However, the relationship is not so straightforward and an inverse effect has been seen with WEEV-infected *Cx. tarsalis* mosquitoes and CHIKV-infected *Ae. albopictus* mosquitoes [267,268].

A mechanistic explanation for the effect of temperature on viral infection was proposed by Adelman et al. These authors found that although siRNA production was unaffected, RNAi efficiency was lower when *Ae. aegypti* mosquitoes were reared at a cooler temperature of 18 °C, which in turn could have led to increased replication of CHIKV and YFV in these mosquitoes [269]. The authors hypothesized that the differential effect of low temperature on virus replication and the RNAi machinery determines the eventual outcome on virus transmission [269,270].

Several studies have shown that large fluctuations in diurnal temperatures at a higher mean temperature (26°C–30°C) have a detrimental impact on development of immature stages, fecundity, survival and susceptibility to DENV infection by prolonging the EIP [271,272,273]. These results correlate well with the seasonal variation associated with DENV transmission in an endemic area like Thailand, where smaller variations in temperature are seen during the high transmission season as opposed to larger fluctuations during the low transmission season [271]. However, the same authors found that similar fluctuations at a lower mean temperature (20 °C) result in shorter EIPs and increased potential for DENV transmission [272]. It is thus evident that variation induced by temperature has a significant effect on vector competence. This environmental variable is multi-factorial and given the importance of climate change on the global spread of mosquitoes and arboviruses, it is paramount to model its effect on virus transmission using realistic and natural parameters.

### 6.2. Sexual Dimorphism and Mating

Sexual dimorphism in immunocompetence is common across animals, where there is a trend towards males suffering higher costs of infection [274]. In *Drosophila*, this may be true of viral infections, which seem to cause more harm in males (although female flies are more affected by bacterial infections) [275]. For example, the dicistroviruses DCV and CrPV are associated with higher mortality rates in males, and DCV grows to a higher titer, indicating that males are less resistant to these viruses [55,276]. Additionally, males have a lower tolerance to Kallithea virus infection, and die at a much higher rate than females, even though Kallithea virus replicates to a lower titer in males. Sex-specific immunity has not been well investigated in mosquitoes, because males are not hematophagous and unable to vector viruses of medical concern.

Although reduced male immune function can be theoretically explained by sex-specific investment in mating success and survival, the molecular mechanisms remain unclear, and may be specific to any given host–parasite combination [277,278,279]. Basic differences in immune-related traits between uninfected males and females may, in part, help to explain the observed sexual dimorphism in resistance and tolerance. For instance, females may be more developmentally resilient against infection. There is a higher basal rate of intestinal stem cell proliferation and gut renewal, more circulating phagocytes, and higher phenoloxidase activity in females [275,280,281]. Conversely, males may be more primed for resisting infection on a transcriptional level, and genes encoding for Toll pathway components tend to have higher basal expression in males [275].

In *Drosophila*, mating induces an immunosuppressive state, marked by variable AMP expression and reduced resistance to some bacterial infections [282,283,284,285]. Although this may occur in other insect species as well [286], it has not been well-studied in mosquitoes, presumably because blood-feeding is contingent on copulation. The reduction in immune function in female flies has been linked with increased synthesis of juvenile hormone (JH) [285,287,288], a multifunctional hormone required for oogenesis [289]. JH signals through the germ cells expressed (gce) receptor, and is itself activated by a male seminal protein, Sex Peptide (SP), that is transferred to the female during mating [290]. Removal of the corpus allatum (the endocrine gland where JH is synthesized) or genetic ablation of *gce* or *JH* in female flies, or *SP* in male flies, restored virgin levels of immunity post-copulation [285]. Although the effect of mating on antiviral immune function has not been studied in flies, a *Heliothis zea* (the corn earworm) nudivirus and a *Mythimna separata* (the northern armyworm) entomopoxvirus encode JH regulatory genes likely obtained by horizontal transfer, indicating JH synthesis may be beneficial to some DNA viruses [291,292].

### 6.3. Infection History

Infection with other viruses in the past and present may also influence the efficacy of antiviral responses or replication of cohabiting viruses. Co-infection may occur frequently in *D. melanogaster*, although interactions between viruses in this host have not been tested. In a Japanese population of singly collected flies, 42% were uninfected, 13% were infected with a single virus, whereas 19% had two infections, and the rest had multiple infections (*n* = 31) [293]. A similar pattern has been observed in honey bees [294]. This could either imply that there are flies with general susceptibility to diverse viruses, or that virus-by-virus interactions can promote infection in some cases, possibly through expression of virally-encoded immune inhibitors [295,296]. A consistent observation has been described in *Cx. tritaeniorhynchus* cells persistently infected with Culex flavivirus (CxFV), which led to slight enhancement in Japanese encephalitis virus (JEV) and DENV replication [297].

In areas endemic for arbovirus transmission, multiple arboviruses may be co-circulating at the same time. There have been several reports of DENV and CHIKV co-infections in humans during these outbreaks [298,299,300]. Recently, with outbreaks of DENV, CHIKV and ZIKV coinciding in Latin America, cases of triple co-infection have been detected [301]. The potential of *Ae. aegypti* and *Ae. albopictus* mosquitoes to simultaneously transmit both or all three viruses in their saliva was investigated by different groups. Indeed, multiple studies showed that a combination of two different viruses or all three could be transmitted at the same time in the mosquito saliva. Only minor interference effects were detected due to co-infection or infection of one virus prior to another [302,303,304]. As such, co-infection or dual infection of *Aedes* spp. mosquitoes with other co-circulating arboviruses do not seem to influence vector competence.

Alternatively, superinfection exclusion has been described in mosquito cells infected by mosquito-specific viruses (i.e., non-vectored mosquito viruses), whereby a virus-infected cell is refractory towards a secondary infection by a similar virus, although by an unknown mechanism [183,305]. For example, prior infection of *Ae. albopictus* C6/36 cells by Palm Creek virus (PCV) was found to moderately suppress the replication of two different flaviviruses—Murray Valley encephalitis virus and WNV [306]. In *Cx. annulirostris*, high PCV titers could suppress subsequent WNV infection when the virus was introduced through an infectious blood meal, although not when intra-thoracically inoculated. PCV was found to localize to mosquito midgut cells and may potentially interfere with midgut infection or escape [307]. Similarly, the insect-specific Nhumirim virus (NHUMV) was found to significantly interfere with the replication of WNV and SLEV in C6/36 cells, and to reduce WNV transmission by 40% in *Cx. quinquefasciatus* in vivo [308,309]. Finally, Eilat virus (EILV), a mosquito-specific alphavirus, interfered with a panel of alphaviruses in vitro, and delayed CHIKV infection in *Ae. aegypti* [310], similar to early CxFV inhibition of WNV in *Cx. pipiens* [311]. Despite considerable evidence that mosquito-specific viruses can modulate vector competence of several mosquito species, world-wide distributions and frequencies of co-infections are unknown, and no association of these viruses with refractory or susceptible populations of a particular mosquito species has been found.

Recently, a mechanism for antiviral immune memory has been described in flies, hinting that past infections could contribute to variation in defense among individuals. During infection, hemocytes take up virus and exogenous viral dsRNA, which are reverse transcribed into DNA molecules [23,24,25]. These vDNAs serve as a template for de novo synthesis of secondary siRNA, which are packaged into exosomes and are proposed to mediate a systemic immune response [25]. Although virus-specific immune memory has not been observed for DCV infections [312], immune memory has been observed in the Indian mealmoth, *Plodia interpuctella,* in response to a DNA virus, where the memory benefit can be trans-generational [313]. Therefore, successful mitigation of virus infection may render the host refractory towards future infection with similar strains.

### 6.4. Endogenous Viral Elements—Non-Retroviral Integrated RNA Virus Sequences

Arboviruses are non-retroviral positive or negative-sense RNA viruses that do not produce a DNA intermediate in their replication cycle. Surprisingly, however, arbovirus-derived viral DNA (vDNA) forms have been detected in infected mosquitoes and mosquito cells [314,315]. These vDNA forms are thought to be byproducts of reverse transcriptase activity by cellular retrotransposons [23,24]. Indeed, treating infected cells and mosquitoes with azidothymidine (a reverse transcriptase inhibitor) led to a dose-dependent reduction in vDNA and reduced production of virus-derived siRNAs in CHIKV-infected *Ae. albopictus* mosquitoes. Most importantly, loss of vDNAs reduced the survival of infected mosquitoes, suggesting that vDNA production is essential for tolerance to infection, possibly by modifying small RNA production [24]. If confirmed in other virus–vector combinations, these results suggest that a complex interplay between viruses, transposable elements, vDNA production and RNAi responses are crucial for sustaining a stable infection and thus for transmission to a susceptible mammalian host.

During the course of acute infection, vDNAs were also found in the wings and legs of infected mosquitoes, suggesting that these DNA elements may be episomal [24]. Although no experimental evidence exists at the moment, it is quite possible that these viral DNA forms are the precursors to endogenous viral elements (EVEs, also referred to as non-retroviral integrated RNA virus sequences), found integrated in the genomes of several vector mosquito species [314,316,317,318,319]. Recently, a comprehensive bioinformatic analysis revealed EVEs from different arboviral families (*Flaviviridae*, *Rhabdoviridae*, *Reoviridae* and members from the Order *Bunyavirales*) predominantly in the genomes of *Ae. aegypti* and *Ae. albopictus* and to a much lower extent in *Cx. quinquefasciatus* and Anopheline mosquitoes [320]. Most of these sequences belong to insect-specific viruses, which are likely maintained in nature by transovarial transmission and would therefore be more likely to infect germline cells, produce viral DNA forms, and be stably inherited [320]. EVEs were found to be significantly enriched in piRNA clusters, but were also found in genic and intergenic regions. More importantly, EVEs produced piRNAs in an antisense orientation to viral mRNA sequences, suggesting that they have antiviral potential. There is variation in the EVE content in the genomes from geographic populations of mosquitoes, suggesting that the acquisition of EVEs is a highly variable process and that the EVE repertoire could be an archive of past infections [320]. An exciting possibility is that integration of viral sequences in mosquito genomes coupled with their production of antisense piRNAs serve as an RNAi-based adaptive immune system and could potentially either target or help tolerate infections with cognate viruses [321]. These new insights into the dynamic composition of mosquito genomes coupled with the compelling phenotype of vDNA-associated tolerance provide a new perspective on the molecular basis for variation in virus–vector interactions.

## 7. Concluding Remarks

The outcome of viral infection is characterized, in part, by general immune responses, virus-specific responses, and variation among host individuals. The mechanisms and relative importance of these factors in host–virus interactions have been the focus of many studies on *Drosophilidae* and *Culicidae* insects and their infecting viruses. *D. melanogaster* has been an instrumental model in discerning molecular mechanisms of immunity, and large infection experiments across genotypes have led to the identification of antiviral resistance loci. However, only five natural viruses have been isolated, and, of these, three have been used in genome-wide association studies (GWAS) or mapping studies. GWAS for resistance to non-natural viruses found no associations, underlining the importance of shared coevolutionary history in maintaining genetic variation for resistance [130]. Thus, the isolation of new *Drosophila* viruses will enable a more powerful comparative framework to assess the generality of immune responses and patterns of genetic variation in resistance. In contrast, there are many isolates of native mosquito viruses, but very few studies focusing on identifying host loci underlying resistance to viral infection. Although large-effect QTL have been mapped for DENV and LACV transmission (Table 2), difficult lab rearing, a lack of genomic tools, highly structured populations, and an incomplete understanding of arbovirus host range has largely precluded fine scale mapping experiments or GWAS of viral resistance (Table 2) [10,137]. The recent release of the complete *Ae. aegypti* genome marks an important step in overcoming these hurdles, and may make quantitative genetics in this species more feasible [322]. Comparative transcriptomic studies in *Ae. aegypti* populations susceptible and refractory to DENV infection have led to the identification of a few host genes that may contribute to differential vector competence [184,185]. However, many of these genes need to be validated in multiple susceptible versus refractory populations and their precise molecular mechanisms need to be characterized. The further implementation of gene-knockout techniques in live mosquitoes will undoubtedly shed further light on the role of these factors in determining vector competence.

## Figures and Tables

**Table 1:** Viruses discussed in this review.

Virus Name	Abbreviation	Genome	Family	Host Restriction
Cricket paralysis virus	CrPV	(+) ssRNA	Dicistroviridae	Insect-specific
Drosophila C virus	DCV	(+) ssRNA	Dicistroviridae	Insect-specific
Culex flavivirus	CxFV	(+) ssRNA	Flaviviridae	Insect-specific
Nhumirim virus	NHUMV	(+) ssRNA	Flaviviridae	Insect-specific
Palm Creek virus	PCV	(+) ssRNA	Flaviviridae	Insect-specific
Flock House virus	FHV	(+) ssRNA	Nodaviridae	Insect-specific
Eilat virus	EILV	(+) ssRNA	Togaviridae	Insect-specific
Nora virus	Nora	(+) ssRNA	Unclassified Picornavirales	Insect-specific
Drosophila melanogaster sigma virus	DmelSV	(−) ssRNA	Rhabdoviridae	Insect-specific
Drosophila X virus	DXV	dsRNA	Birnaviridae	Insect-specific
Invertebrate iridescent virus 6	IIV6	dsDNA	Iridoviridae	Insect-specific
Kallithea virus	Kallithea	dsDNA	Nudiviridae	Insect-specific
Dengue virus	DENV	(+) ssRNA	Flaviviridae	Arbovirus
Japanese encephalitis virus	JEV	(+) ssRNA	Flaviviridae	Arbovirus
Murray Valley encephalitis virus	MVEV	(+) ssRNA	Flaviviridae	Arbovirus
St Louis encephalitis virus	SLEV	(+) ssRNA	Flaviviridae	Arbovirus
West Nile virus	WNV	(+) ssRNA	Flaviviridae	Arbovirus
Yellow fever virus	YFV	(+) ssRNA	Flaviviridae	Arbovirus
Zika virus	ZIKV	(+) ssRNA	Flaviviridae	Arbovirus
Chikungunya virus	CHIKV	(+) ssRNA	Togaviridae	Arbovirus
O’nyong’nyong virus	ONNV	(+) ssRNA	Togaviridae	Arbovirus
Semliki Forest virus	SFV	(+) ssRNA	Togaviridae	Arbovirus
Sindbis virus	SINV	(+) ssRNA	Togaviridae	Arbovirus
Venezuelan equine encephalitis virus	VEEV	(+) ssRNA	Togaviridae	Arbovirus
Western equine encephalitis virus	WEEV	(+) ssRNA	Togaviridae	Arbovirus
La Crosse encephalitis virus	LACV	(−) ssRNA	Peribunyaviridae	Arbovirus
Rift Valley fever virus	RVFV	(−) ssRNA	Phenuiviridae	Arbovirus
Vesicular stomatitis virus	VSV	(−) ssRNA	Rhabdoviridae	Arbovirus

**Table 2:** Genetic variants associated with viral resistance.

Host Factor	Virus	Host	Associated Mutation	Phenotype (Homozygotes)	Population Frequency (Resistant Allele)	Related Processes	Methodology	References
*ref(2)P*	DmelSV	Dmel	NS polymorphism	24% reduction in infection rate	24%	Autophagy	Genetic mapping, mutagenesis, GWAS	[127,128,129,130]
*CHKov1/CHKov2*	DmelSV	Dmel	TE insertion, rearrangement	52% reduction in infection rate	TE: 83%; rearrangement <0.5%	Predicted acetylcholine esterase	Genetic mapping, GWAS	[130,131]
*Ge-1*	DmelSV	Dmel	26 aa deletion	97% reduction in infection rate	1%	RNA decay	Genetic mapping	[40]
*ref(1)H QTL*	DmelSV	Dmel	Unknown	Unknown	Unknown	Unknown	Genetic mapping	[128]
*ref(3)O QTL*	DmelSV	Dmel	Unknown	Unknown	Unknown	Unknown	Genetic mapping	[128]
*ref(3)V QTL*	DmelSV	Dmel	Unknown	Unknown	Unknown	Unknown	Genetic mapping	[128]
*X13 QTL*	DmelSV	Dmel	Unknown	38% reduction in infection rate	Unknown	Unknown	Genetic mapping	[132]
*X65 QTL*	DmelSV	Dmel	Unknown	13% reduction in infection rate	Unknown	Unknown	Genetic mapping	[132]
*2R70 QTL*	DmelSV	Dmel	Unknown	11% reduction in infection rate	Unknown	Unknown	Genetic mapping	[132]
*3R64 QTL*	DmelSV	Dmel	Unknown	12% reduction in infection rate	Unknown	Unknown	Genetic mapping	[132]
*pastrel*	DCV	Dmel	7 alleles: cis-regulatory, structural, NS variants	16%, 57%, or 80% increase in survival	NS: 7–33%, Structural variants: 4–51%	Unknown	GWAS, experimental evolution	[130,133,134]
*2R69 QTL*	DCV	Dmel	Unknown	2.5 day increase in survival time	Unknown	Unknown	Recombinant inbred line	[132]
*2L18 QTL*	DCV	Dmel	Unknown	0.75 day increase in survival time	Unknown	Unknown	Recombinant inbred line	[132]
*APC7*	DCV	Dmel	Synonymous polymorphism	95% increase in survival	3%	Cell cycle	GWAS	[130]
*Ubc-E2H*	DCV	Dmel	Intronic polymorphism	Not reported	27% (lab-maintained population)	Predicted ubiquitin ligase	Experimental evolution	[133]
*Cip4*	Kallithea	Dmel	NS polymorphism	27% increase in survival	77%	Membrane trafficking	GWAS	[107]
*Dicer-2*	DENV-1	Aaeg	Unknown	Explains 17.8% of viral dissemination	Unknown	RNAi	Genetic mapping	[135]
*71CGT1 QTL*	DENV-1/3	Aaeg	Unknown	QTL explains up to 7.6% variation in MIB	Unknown	Unknown	Genetic mapping	[136]
*335CGA1 QTL*	DENV-1/3	Aaeg	Unknown	QTL explains ≤ 8.1% variation in MIB and ≤21.4% in dissemination	Unknown	Unknown	Genetic mapping	[136]
*88CA1/88GAA1 QTL*	DENV-1/3	Aaeg	Unknown	QTL explains ≤ 12% variation in MIB and ≤75.6% in titer	Unknown	Unknown	Genetic mapping	[136]
*301CT1/301ACG1 QTL*	DENV-1/3	Aaeg	Unknown	QTL explains ≤ 11.4% variation in MIB	Unknown	Unknown	Genetic mapping	[136]
*B19 QTL*	DENV-1/3	Aaeg	Unknown	QTL explains ≤ 6.2% variation in MIB	Unknown	Unknown	Genetic mapping	[136]
*69TGA1 QTL*	DENV-1/3	Aaeg	Unknown	QTL explains ≤ 22.6% variation in dissemination and ≤ 8.9% in titer	Unknown	Unknown	Genetic mapping	[136]
*201AAT1 QTL*	DENV-1/3	Aaeg	Unknown	QTL explains ≤ 15.2% variation in titer	Unknown	Unknown	Genetic mapping	[136]
*470CT2/470AG1 QTL*	DENV-1/3	Aaeg	Unknown	QTL explains ≤ 12.3% variation in titer	Unknown	Unknown	Genetic mapping	[136]
*17ATA1 QTL*	DENV-1/3	Aaeg	Unknown	QTL explains ≤ 13.7% variation in titer	Unknown	Unknown	Genetic mapping	[136]
*early trypsin* QTL	DENV-2	Aaeg	Unknown	86% decrease in infection dissemination	Unknown	Blood meal digestion	Genetic mapping	[137]
*B18.621* QTL	DENV-2	Aaeg	Unknown	87% decrease in infection dissemination	Unknown	Unknown	Genetic mapping	[137]
*B20.392* QTL	LACV	Otri	Unknown	Transovarial transmission rate increased from 0 to 60%	Unknown	Unknown	Genetic mapping	[138]
*C01.385/C13.5573* QTL	LACV	Otri	2 linked QTL with unknown mutations	Transovarial transmission rate increased from 0 to 60%	Unknown	Unknown	Genetic mapping	[138]

Dmel, *D. melanogaster*; Aaeg, *Ae. aegypti*; Otri, *O. triseriatus*; aa, amino acid; MIB, midgut infection barrier; NS, nonsynonymous; S, synonymous; TE, transposable element; GWAS, genome-wide association study.
